# Crosstalk Between N6-Methyladenosine and Other Epigenetic Mechanisms in Central Nervous System Development and Disorders

**DOI:** 10.3390/biom15081092

**Published:** 2025-07-28

**Authors:** Cuiping Qi, Xiuping Jin, Hui Wang, Dan Xu

**Affiliations:** 1 Department of Obstetrics, Zhongnan Hospital of Wuhan University, School of Pharmaceutical Sciences, Wuhan University, Wuhan 430071, China; qcp20192345@whu.edu.cn (C.Q.); 2022203060053@whu.edu.cn (X.J.); 2 Department of Pharmacology, Taikang Medical School (School of Basic Medical Sciences), Wuhan University, Wuhan 430071, China; wanghui19@whu.edu.cn; 3 Hubei Provincial Key Laboratory of Developmentally Originated Disease, Wuhan 430071, China

**Keywords:** epigenetic modifications, N6-methyladenosine, central nervous system, reprogramming regulation, development and diseases

## Abstract

A variety of epigenetic mechanisms—such as DNA methylation, histone alterations, RNA chemical modifications, and regulatory non-coding RNAs—collectively influence gene regulation and cellular processes. Among these, N6-methyladenosine (m^6^A) represents the most widespread internal modification in eukaryotic mRNA, exerting significant influence on RNA metabolic pathways and modulating mRNA function at multiple levels. Studies have shown that m^6^A modification is highly enriched in the brain and regulates central nervous system development and various physiological functions. Recent studies have demonstrated that m^6^A interacts with other epigenetic regulators and triggers epigenetic remodeling, which further affects the development and occurrence of central nervous system diseases. In this review, we provide an up-to-date overview of this emerging research hotspot in biology, with a focus on the interplay between m^6^A and other epigenetic regulators. We highlight their potential roles and regulatory mechanisms in epigenetic reprogramming during central nervous system development and disease, offering insights into potential novel targets and therapeutic strategies for CNS disorders.

## 1. Introduction

**Figure 1:**
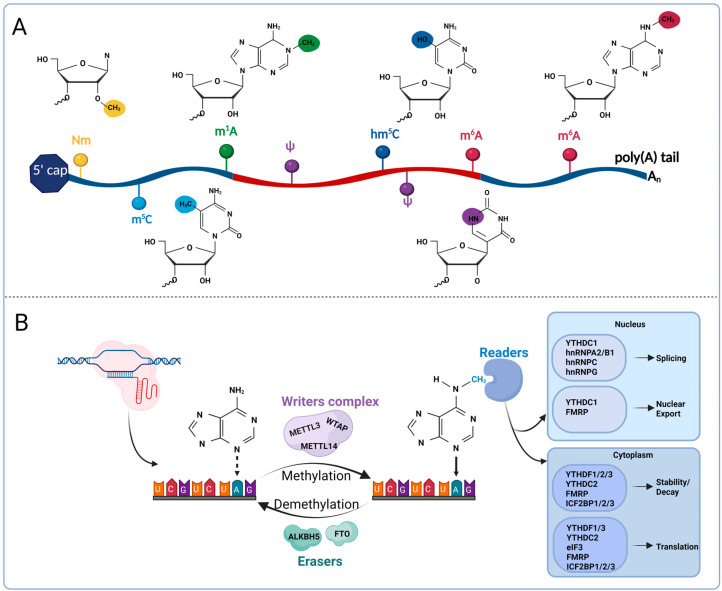
Common forms of RNA methylation and m^6^A-mediated mRNA regulation. (**A**) Illustrated here are N^6^-methyladenosine (m^6^A, red), pseudouridine (ψ, purple), 2′-O-methylation (Nm, gold), 5-methylcytosine (m^5^C, cyan), N^1^-methyladenosine (m^1^A, green), and 5-hydroxymethylcytosine (hm^5^C, navy blue). Each modification is depicted at its representative position on a simplified RNA strand. To emphasize their widespread and functionally diverse distribution across mRNAs, tRNAs, rRNAs, and snRNAs, m^6^A and ψ are shown more than once along the RNA strand. In contrast, other modifications are displayed once at their most canonical or functionally relevant site. (**B**) Methylation of mRNA substrates with m^6^A is catalyzed by writers, with the best-characterized writer being a multi-subunit complex consisting of the core enzymes METTL3, METTL14, and WTAP. The reverse process of demethylation is mediated by two erasers, FTO and ALKBH5. m^6^A-modified mRNAs are recognized by a diverse group of RNA-binding proteins called readers, which regulate several aspects of mRNA metabolism, including splicing, nuclear export, decay, stability, and translation.

Epigenetic regulation involves transmissible changes in gene activity that occur without altering the underlying DNA sequence. These modifications typically arise from chemical alterations to DNA or chromatin-associated proteins. Key epigenetic mechanisms include DNA methylation, histone tail modifications, regulatory non-coding RNAs (ncRNAs), and various RNA chemical marks. Among these, RNA modifications are particularly widespread in eukaryotic cells, with more than 170 types identified to date [1]. Notably, RNA methylation represents the majority, comprising approximately two-thirds of all RNA modifications. Common types include N6-methyladenosine (m^6^A), 5-methylcytosine (m^5^C), 1-methyladenosine (m^1^A), 5-hydroxymethylcytosine (hm^5^C), pseudouridine (ψ), and 2′-O-methylation (Nm) (Figure 1A) [2,3]. Among them, m^6^A, first reported in 1974, is the most prevalent methylation mark in eukaryotic RNA, constituting around 80% of total RNA methylation events [4,5]. It participates in regulating multiple biological functions of RNA, but was only recently studied due to the limitations of early technology [6,7,8]. The regulation of m^6^A mainly depends on the methyltransferase complex, demethylases, and methyl-binding proteins, which dynamically adjust the modification through demethylation [9,10]. The interaction between m^6^A and other epigenetic regulators has been increasingly recognized as a key contributor to epigenetic remodeling, further adding to the complexity of epigenetic mechanisms. This is currently a hot research topic.

Central nervous system (CNS) development is a highly coordinated process that requires regulation at multiple levels, including epigenetic modifications, to ensure the formation of normal functions. Sun et al. and Huang et al. demonstrated that m^6^A is highly enriched in the mammalian brain and plays essential roles in regulating early central nervous system (CNS) development as well as maintaining physiological functions in adulthood [11,12]. For example, m^6^A-mediated gene regulation has been demonstrated to be a crucial step in neural cell differentiation, and m^6^A controls the self-renewal and differentiation of embryonic stem cells in mammals [13]. However, the role of m^6^A in adverse events in the CNS is still poorly understood. The crosstalk between m^6^A and other epigenetic regulators increases the complexity of epigenetic reprogramming and involves multiple aspects, including CNS development and neurological and psychiatric disorders [9].

It is important to critically acknowledge that this research field still faces substantial limitations and knowledge gaps. For instance, the functional consequences of specific m^6^A modification sites remain poorly understood, and there is currently a lack of standardized tools that enable the precise manipulation of m^6^A levels at defined loci [14]. Although several small-molecule inhibitors, such as FTO inhibitors, have demonstrated therapeutic potential in preliminary studies, their target specificity, bioavailability, and long-term safety have not yet been fully validated. Moreover, existing studies are predominantly focused on developmental stages or cancer biology, with relatively limited systematic investigation into the role of m^6^A in specific neuropsychiatric or neurodegenerative diseases. Finally, the in vivo bidirectional regulatory mechanisms between m^6^A and other epigenetic layers—such as histone modifications and non-coding RNAs—remain largely unexplored, which constrains the broader application of m^6^A research in the context of epigenetic reprogramming.

This review systematically summarizes recent progress in this emerging field, with a particular focus on the interplay between m^6^A and other epigenetic mechanisms. It further highlights the potential roles and regulatory pathways of m^6^A and its modulators in mediating epigenetic reprogramming during neurodevelopment and disease pathogenesis.

## 2. Molecular Regulatory Mechanisms and Biological Functions of m^6^A

### 2.1. Molecular Regulatory Mechanisms of m^6^A

The regulation of m^6^A modification is mediated through the interplay of three classes of proteins, methyltransferases (“writers”), demethylases (“erasers”), and m^6^A-binding proteins (“readers”) [15], which, together, determine the methylation status and functional outcomes of target transcripts (Figure 1B).

The installation of m^6^A marks on RNA is predominantly carried out by a methyltransferase complex, in which methyltransferase-like 3 (METTL3) functions as the enzymatic core. This writer complex also comprises essential cofactors, including methyltransferase-like 14 (METTL14), which facilitates RNA substrate recognition, and Wilms tumor 1-associating protein (WTAP), which contributes to the localization and stability of the complex [16]. METTL3 and METTL14 form a stable heterodimer, which is central to both substrate recognition and methyl group transfer [13]. WTAP, in turn, interacts with this core dimer to ensure proper subnuclear localization within nuclear speckles and to facilitate full methylation activity [17]. Notably, WTAP depletion impairs the RNA-binding ability of METTL3 [18]. Beyond these core elements, several accessory proteins—such as VIRMA (also known as KIAA1429), ZC3H13, RBM15, and CBLL1—associate with the complex to refine methylation site selection and modulate transcription-linked signaling pathways [19,20].

The identification of demethylases has revealed that m^6^A modification is a reversible and dynamically regulated process. Two key enzymes responsible for m^6^A demethylation are fat-mass- and obesity-associated protein (FTO) and AlkB homolog 5 (ALKBH5). FTO has been shown to remove both m^6^A and N6,2′-O-dimethyladenosine (m^6^Am) modifications from mRNA, with a markedly higher catalytic preference—approximately 100-fold—for m^6^Am over m^6^A [21,22,23]. However, recent data do not support FTO as an eraser of m^6^A, and indicate that FTO does not remove m^6^A under normal physiological conditions, but only acts on m^6^Am [22,24,25]. Therefore, the removal of m^6^A by FTO may be the result of environmental and cell-type-specific regulation. ALKBH5 reverses m^6^A by oxidation, affecting mRNA output, metabolism, and the assembly of mRNA processing factors in nuclear speckles [26,27]. In summary, knocking down FTO and ALKBH5 in cell lines significantly increases the level of m^6^A modifications on mRNA.

A specific RNA-binding protein (RBP) is required for mRNA with m^6^A modification to perform specific biological functions, commonly found in the conserved YT521-B homologous domain (YTH) family of proteins, including YTH domain family proteins (YTHDF) 1, 2, and 3 and YTH-domain-containing proteins (YTHDC) 1 and 2, which bind to m^6^A-modified RNA through the m^6^A binding domain [28,29]. YTH-domain-containing proteins are key mediators of m^6^A function, influencing various aspects of mRNA metabolism, including splicing, nuclear export, translation, and degradation [30,31]. In addition to YTH proteins, other RNA-binding proteins (RBPs) can recognize m^6^A-modified transcripts through conventional RNA-binding domains. These include heterogeneous nuclear ribonucleoproteins (hnRNPs) [32], fragile X mental retardation protein (FMRP) [33], insulin-like growth factor 2 mRNA-binding proteins (IGF2BPs) [34], and eukaryotic initiation factor 3 (eIF3) [33], among others. Interestingly, recent findings suggest that certain RBPs, such as G3BP1 and G3BP2, may be repelled by m^6^A marks, thereby adding further complexity to m^6^A-mediated post-transcriptional gene regulation [35].

### 2.2. Biological Functions of m^6^A

m^6^A can affect a wide range of downstream functions, including RNA splicing and output from the nucleus, as well as the translation, degradation, and stability regulation of mRNA in the cytoplasm (Figure 1B).

m^6^A can regulate RNA splicing and output through various mechanisms. In the nucleus, m^6^A splicing is mediated by several binding proteins, which regulate the selective splicing of target transcripts by directly binding to YTH-domain-containing proteins such as YTHDC1 [36], or by relying on m^6^A structural switches such as hnRNPC and hnRNPG [37]. In terms of nuclear output, YTHDC1 promotes m^6^A-modified transcript output mediated by nuclear RNA export factor 1 (NXF1) through interaction with SRSF3 [38], while FMRP binds to m^6^A-modified RNA and promotes its nuclear output through exportin 1 (CRM1) [39]. Although m^6^A modification has an unstable effect on target RNA, a positive correlation has been observed between m^6^A and mRNA translation efficiency. In the cytoplasm, YTHDF1 and YTHDF3 bind near the stop codon of mRNA, promoting translation [40,41]. YTHDF1 has been shown to enhance mRNA translation by directly associating with the eIF3 complex [42,43]. Additionally, m^6^A modifications themselves can facilitate cap-independent translation through a direct interaction with eIF3, independent of YTH proteins [44]. However, emerging evidence has questioned the traditional classification of YTHDF1 and YTHDF3 as translation enhancers. Instead, it has been proposed that all three YTHDF family members may primarily contribute to mRNA degradation driven by m^6^A modification [6]. Among the YTHDF proteins, YTHDF2 appears to play the most prominent role in promoting mRNA decay. It can mediate degradation through multiple mechanisms: by directing m^6^A-tagged transcripts to cytoplasmic processing bodies (P-bodies) [30], by recruiting the CCR4-NOT deadenylase complex to initiate degradation independently of P-bodies [45], or by cooperating with adaptor protein HRSP12 and ribonuclease RNase P/MRP to cleave target RNAs [46]. Conversely, some proteins have been identified that stabilize m^6^A-modified transcripts. For instance, FMRP has been reported to bind competitively with YTHDF2 to shared m^6^A-marked targets, thereby preventing their degradation and supporting transcript stability [47]. In addition, IGF2BP1-3 have also been shown to stabilize mRNA expression by recognizing m^6^A-marked mRNA [48,49].

## 3. The Role of m^6^A in the Development of the Central Nervous System

**Figure 2:**
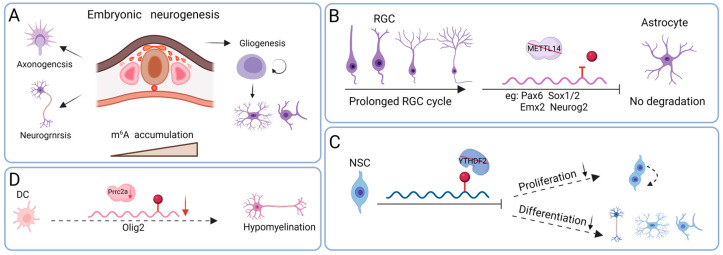
The role of m^6^A in brain development. (**A**) m^6^A exhibits a progressive increase throughout brain development, contributing to the regulation of axon formation, neuronal differentiation, and glial cell lineage commitment. (**B**) The conditional deletion of METTL14 in embryonic mouse brains abolishes m^6^A deposition and leads to an extended radial glial cell cycle. (**C**) YTHDF2 deficiency in neural stem cells impairs both proliferative capacity and neurogenic potential. (**D**) Proline-rich coiled-coil 2A (PRRC2A) enhances the stability of oligodendrocyte transcription factor 2 (*Olig2*) mRNA—a critical transcription factor in oligodendrocyte development—by binding to its m^6^A-modified coding sequence. The conditional knockout of *Prrc2a* results in pronounced deficits in myelination.

m^6^A is highly abundant in the mammalian brain and serves as a critical regulator of neural development, contributing to the regulation of various processes, such as neurogenesis, glial cell formation, and axonal development (Figure 2A).

### 3.1. Neurogenesis

Neurogenesis is a multifaceted biological process wherein neural stem cells proliferate, differentiate into lineage-specific progenitors, migrate to target brain regions, undergo structural and functional remodeling, and, ultimately, form synaptic connections essential for neural circuit integration. Yoon et al. revealed that m^6^A plays an important role in regulating cortical neurogenesis [50]. Specifically, the loss of METTL14 in the E13.5 mouse cortex led to a downregulation of m^6^A modification on transcripts related to the cell cycle, stem cell maintenance, and neuronal differentiation, resulting in the prolonged cell cycle progression of radial glia cells (RGCs) (Figure 2B) [50]. This suggests that m^6^A can mediate cell cycle exit, which is necessary for maintaining normal cortical neurogenesis. Neuroglial cells (NSCs) lacking YTHDF2 exhibit decreased proliferation and differentiation, which is associated with transcripts regulating the JAK-STAT pathway. Neurons originating from these NSCs exhibit reduced neurite outgrowth and heightened vulnerability to oxidative stress (Figure 2C) [51]. Moreover, the loss of YTHDF2 markedly disrupts the self-renewal capacity of embryonic cortical neural progenitor cells and impairs neurogenesis. This disruption correlates with the upregulation of m^6^A-modified transcripts involved in neurodevelopment and cortical neuronal differentiation, underscoring the critical role of YTHDF2-dependent mRNA decay in orchestrating cortical neurogenesis during embryogenesis.

### 3.2. Gliogenesis

NSCs are the stromal cells of the nervous system, characterized by processes but without dendrites and axons, accounting for about 90% of the total cells in the CNS, and mainly play roles in repair and regeneration, support, the maintenance of substance metabolism, and nutrition. In YTHDF2- and METTL14-deficient NSCs, it has been shown that NSCs cannot differentiate into astrocytes, affecting the generation of astrocytes (Figure 2C) [50,51]. The novel m^6^A-binding protein proline-rich coiled-coil 2A (PRRC2A) regulates the proliferation of oligodendrocyte progenitor cells and the generation of oligodendrocytes. PRRC2A binds to the CDS of *Olig2* mRNA with the GGACU motif of m^6^A modification, resulting in a reduction in oligodendrocyte myelin formation and inducing motor and cognitive defects (Figure 2D) [52]. In addition, the loss of the METTL14 gene in oligodendrocytes can lead to a reduction in specific neurofascin (NfASC) splice variants, causing abnormal Ranvier node morphology and the abnormal splicing of the paranodal antibody–neurofascin 155 (NF155) transcript, ultimately resulting in a reduction in the number of oligodendrocytes [53]. Therefore, m^6^A modification plays a crucial role in neuroglial cell generation.

### 3.3. Axonal Growth

Axons are the primary channels for neuronal signal transmission in the nervous system, which extend along specific pathways over long distances to reach their targets and form precise neural connections. Axonal growth is influenced by various factors, among which guidance molecules with attractive or repulsive effects play a major role. m^6^A can affect the mRNA translation involved in axonal guidance and extension. Qi et al. reported that FTO, a demethylase, and its binding protein YTHDF1, respectively, bind to axonal RNAs, suggesting their potential involvement in the regulation of axonal mRNA metabolism [54]. FTO promotes *Gap43* translation via demethylation in the axons of dorsal root ganglion (DRG) neurons, thereby facilitating axonal elongation. In the dorsal spinal cord (DSC), YTHDF1 recognizes methylated axon-guidance-related mRNA *Rono3.1* and promotes its translation, thereby regulating axonal guidance [55]. Recent studies have demonstrated that both YTHDF1 and YTHDF2 are present in cerebellar granule cells and their axonal projections [26]. Silencing either of these m^6^A reader proteins in granule cell axons leads to enhanced parallel fiber elongation and increased cerebellar synaptogenesis. This effect appears to be linked to the local translation of WNT signaling components—*Dvl1* and *Wnt5a*—regulated by YTHDF1 and YTHDF2, respectively. These findings highlight the pivotal role of m^6^A modification in modulating axonal development.

## 4. m^6^A Regulates Physiological Functions of the Central Nervous System

### 4.1. Behavioral Adaptation

**Figure 3:**
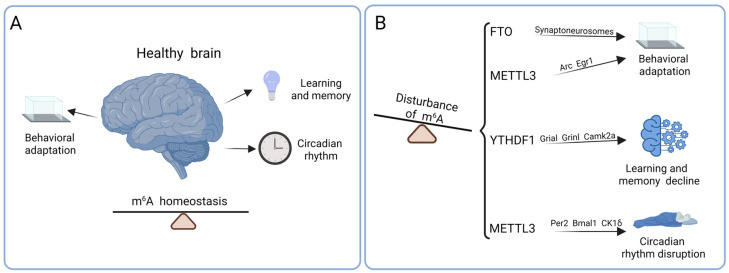
Role of m^6^A in brain physiology. (**A**) m^6^A plays a crucial role in maintaining essential physiological processes such as behavioral adaptation, learning and memory, and circadian rhythm in the adult brain. (**B**) Dysregulation of m^6^A modification can lead to dysfunction of the central nervous system, which may result in various neurological disorders.

Experimental evidence in adult mice indicates that fear-based behavioral training can dynamically modulate m^6^A in the brain. Exposure to both contextual and cued fear conditioning transiently suppresses FTO expression in dorsal hippocampal CA1 neurons and the medial prefrontal cortex, which, in turn, elevates m^6^A levels on a subset of mRNA transcripts [56,57]. The depletion of FTO before training further enhances contextual fear memory (Figure 3B). Under restraint stress conditions, mice with hippocampal METTL3 and FTO deficiencies show lower resilience to stress stimuli, suggesting that m^6^A-mediated mRNA modification may improve stress adaptation [58,59]. m^6^A is also considered crucial for reward mechanisms, as impaired reward stimulation and altered behavioral adaptability are observed in mice lacking FTO [60]. FTO deficiency is a protective factor against chronic stress and relieves depressive and anxiety-like behaviors in FTO knockout mice [61]. However, the absence of FTO, METTL14, or YTHDF1 shows impairments in the cognitive behavior of mice. Overall, abnormal changes in m^6^A can lead to various abnormal neurobehavioral changes, inhibit behavioral adaptability, and induce the occurrence of neuropsychiatric disorders.

### 4.2. Learning and Memory

Several studies on the role of m^6^A in the central nervous system emphasize its importance in learning and memory processes (Figure 3B). For example, METTL3 has a direct role in regulating hippocampus-dependent memory consolidation by promoting the translation of early response genes in neurons [62]. This is related to the increased translation efficiency of early genes mediated by METTL3-mediated m^6^A modification, which is crucial for long-term memory consolidation. Emerging evidence links m^6^A reader proteins to the regulation of learning and memory. Upon neuronal activation, YTHDF1 enhances the translation of m^6^A-modified mRNAs in neurons, facilitating synaptic plasticity and cognitive function. YTHDF1 knockout mice exhibit deficits in learning and memory, accompanied by impaired synaptic transmission and reduced long-term potentiation in the hippocampus [7]. Remarkably, the targeted re-expression of YTHDF1 in the adult hippocampus can rescue these impairments. These findings suggest that the YTHDF1-mediated translational control of m^6^A-tagged transcripts plays a pivotal role in cognitive processes. Thus, m^6^A modification may represent a key epigenetic mechanism underlying memory decline in various neurological disorders.

### 4.3. Circadian Rhythm

The regulation of the 24 h rhythm of biochemistry, physiology, and behavior by the circadian clock is crucial for maintaining normal health status [63]. The circadian clock has been implicated in the pathophysiology of sleep disturbances, neurodegenerative conditions such as Alzheimer’s disease, and various psychiatric disorders including depression. Evidence from animal studies indicates that diminished m^6^A modification on key circadian transcripts—such as *Per1*, *Per3*, *Tef*, *Dbp*, *Nfil3*, *Bhlhe41*, and *Nr1d*—can extend circadian cycles in mice, implying that m^6^A is essential for sustaining clock gene rhythmicity [64]. Specifically, METTL3 deficiency leads to the nuclear retention of unmethylated *Per2* and *Bmal1* transcripts, disrupting the temporal expression of core clock genes (Figure 3B). Moreover, the elimination of the m^6^A-modified region in the *CK1δ* gene, which encodes a kinase involved in clock protein turnover, enhances its translation in the brain and results in prolonged behavioral rhythms in mice [64]. These findings underscore the pivotal role of m^6^A methylation in the regulation of circadian timing mechanisms.

## 5. The Regulation of Central Nervous System Development and Diseases by the Crosstalk Between m^6^A and Other Epigenetic Factors

### 5.1. Inter-Crosstalk Between m^6^A and Other Epigenetic Factors

#### 5.1.1. Inter-Crosstalk Between m^6^A and DNA Methylation

**Figure 4:**
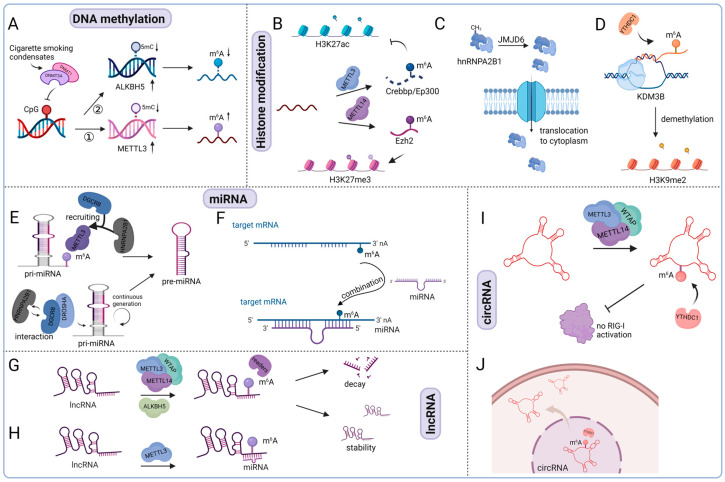
The intricate crosstalk between m^6^A and other epigenetic mechanisms, such as DNA methylation, histone modifications, and non-coding RNAs. (**A**) The loss of 5mC DNA methylation on METTL3 and ALKBH5 promotes their expression. (**B**) m^6^A impacts histone modification by modulating the level of histone-associated enzymes. (**C**) JMJD6 mediates the demethylation of hnRNPA2B1, promoting its translocation to the cytoplasm. (**D**) YTHDC1 activates the m^6^A-labeled chromatin region on KDM3B, triggering H3K9me2 demethylation. (**E**) m^6^A promotes the maturation of miRNAs. (**F**) m^6^A modification triggers the binding of miRNAs to targeted genes. (**G**) m^6^A modulates lncRNA levels. (**H**) m^6^A facilitates the combination of lncRNAs with miRNAs. (**I**) m^6^A modification controls circular RNA immunity. (**J**) m^6^A mediates the cytoplasmic export of circRNAs.

Among various epigenetic mechanisms, DNA methylation is one of the most prevalent in mammals, playing a key role in the regulation of gene expression [65,66]. This modification predominantly occurs at CpG islands within gene promoter regions. In both eukaryotic and prokaryotic genomes, the major forms of DNA methylation are 5-methylcytosine (5mC) and N6-methyladenine (6mA) [67,68,69]. However, the crosstalk between DNA methylation and m^6^A leads to diverse changes in both modifications. m^6^A modification on DNA methyltransferase genes promotes the stability of DNA methyltransferase [70]. Conversely, DNA methyltransferase interacts with m^6^A methyltransferase or demethylase to regulate m^6^A levels. For example, in pancreatic cancer, exposure to cigarette smoke condensate (CSC) has been shown to interfere with the recruitment of DNA methyltransferases DNMT1 and DNMT3A to the METTL3 promoter. This disruption reduces DNA methylation at the METTL3 locus, leading to elevated METTL3 expression and a subsequent increase in global m^6^A levels [71]. In esophageal squamous cell carcinoma, CSC can also cause the low methylation of DNMT1 and DNMT3a in the ALKBH5 CpG island, leading to the low expression of ALKBH5 and the downregulation of m^6^A levels (Figure 4A) [72]. Therefore, there is a wide range of co-transcriptional interferences between m^6^A and DNA methylation modifications.

#### 5.1.2. m^6^A and Histone Modification

Histone modifications encompass several chemical alterations, including acetylation, methylation, phosphorylation, ubiquitination, SUMOylation, and ADP-ribosylation, with methylation and acetylation being the most extensively studied [73]. Methylation typically targets arginine (R) and lysine (K) residues, whereas acetylation predominantly occurs on lysine residues. Both modifications influence gene transcription by either promoting or repressing expression, depending on the specific residues and the context of modification. Studies have found that there is also co-transcriptional interaction between RNA modification and histone modification. For example, the m^6^A methyltransferase complex METTL3-METTL14 interacts with acetyltransferase and methyltransferase to downregulate H3K27ac and upregulate H3K27me3 levels, respectively (Figure 4B). YTHDC1, an m^6^A reader protein, has been shown to associate with the histone demethylase KDM3B, guiding its recruitment to m^6^A-enriched chromatin loci. This interaction facilitates the removal of H3K9me2 marks and enhances the transcription of associated genes (Figure 4D) [74]. Histone modification enzymes can also affect the transport of m^6^A-binding proteins. For instance, after infection with herpes simplex virus-1 (HSV-1), the m^6^A-binding protein Recombinant Mouse Heterogeneous nuclear ribonucleoproteins A2/B1 (hnRNPA2B1) recognizes viral DNA and undergoes homodimerization. Histone demethylase jumanji domain-containing 6 (JMJD6) induces hnRNPA2B1 demethylation, promoting hnRNPA2B1 translocation from the nucleus to the cytoplasm, inducing IFN-α/β production through activating the TBK1-IRF3 pathway and mediating immune response (Figure 4C) [75].

#### 5.1.3. m^6^A and ncRNAs (miRNAs, lncRNAs, and circRNAs)

Non-coding RNAs (ncRNAs), a diverse class of RNA molecules that do not encode proteins, play essential regulatory roles in epigenetic processes. Among them, microRNAs (miRNAs), long non-coding RNAs (lncRNAs), and circular RNAs (circRNAs) have attracted the most attention in epigenetic research [76].

miRNAs are a type of small endogenous RNA, typically 20–24 nucleotides in length. In mammalian systems, the METTL3-mediated methylation of primary miRNAs (pri-miRNAs) enhances their recognition by the RNA-binding protein DGCR8, thereby promoting the efficient processing and maturation of miRNAs (Figure 4E) [77]. Additionally, heterogeneous nuclear ribonucleoprotein A2/B1 (hnRNPA2B1) supports this process by interacting with DGCR8 and facilitating its association with pri-miRNAs, thus contributing to sustained miRNA biogenesis [71,77,78,79]. Since both m^6^A on mRNA and miRNA binding sites are located on the 3′UTR, it is speculated that m^6^A and miRNA may have co-binding effects. Zhang et al. reported that m^6^A modification exists in the 3′UTR of Yes-associated protein 1 (YAP), and this modification promotes the binding of miR-582-3p to YAP [80]. Therefore, m^6^A modification may trigger the binding of miRNA and target genes (Figure 4F). Conversely, miRNA can also control m^6^A levels by inhibiting the expression of m^6^A demethylases [81]. Therefore, there is a bidirectional relationship between miRNA and m^6^A, and the positive and negative feedback loops illustrate the complex relationship between miRNA and m^6^A.

LncRNAs are RNA molecules with lengths generally ranging from 200 to 100,000 nucleotides, some of which fine-tune gene expression through diverse mechanisms in a tissue-specific manner [82]. Recent studies have revealed that the lncRNA regulation of genes is influenced by m^6^A modification, which affects lncRNA degradation and stability, thereby regulating gene expression (Figure 4G) [83,84,85,86]. On the other hand, m^6^A increases lncRNA accessibility to RNA-binding proteins (RBPs) by exposing its purine-rich sequence. Therefore, m^6^A-dependent structural changes in RNA can facilitate the direct binding of m^6^A-modified lncRNA to specific regions of RBP, thereby regulating gene expression [87]. Additionally, m^6^A-binding protein YTHDF3 can interact with lncRNA (Figure 4H) [88]. For example, METTL3-mediated m^6^A modification in support of YTHDF3 contributes to the upregulation of LncRNA MALAT1 expression, thereby enhancing the metastatic potential of non-small cell lung cancer [89].

Unlike conventional linear RNAs, circRNAs possess a covalently closed loop structure that confers resistance to exonuclease-mediated degradation, resulting in enhanced transcript stability [90]. Similar to microRNAs, circRNAs can undergo m^6^A mediated by METTL3 and are recognized by m^6^A reader proteins such as YTHDF1 and YTHDF2, suggesting their involvement in the epitranscriptomic regulation of gene expression [91]. Timoteo et al. revealed that METTL3 regulates m^6^A levels on circRNA, while YTHDC1 affects circRNA splicing. The collaborative regulation of METTL3 and YTHDC1 is involved in various biological processes of circRNA, including translation [92] and splicing [93]. Subsequently, the crosstalk between circRNA and m^6^A modification has been confirmed in numerous diseases. For instance, in patients with colorectal cancer liver metastasis, circNSUN2 is recognized by the m^6^A-binding protein, facilitating its transport from the nucleus to the cytoplasm, increasing the stability of high mobility group A2 (HMGA2), and promoting colon cancer metastasis (Figure 4J) [94]. The m^6^A modification of circular RNAs also plays a role in modulating physiological responses, including immune regulation. Exogenously introduced circRNAs have been shown to function as potent adjuvants, promoting antigen-specific T cell activation, antibody production, and antitumor immunity. However, the recognition of m^6^A-modified circRNAs by the m^6^A reader protein YTHDF2 can suppress these immune responses, indicating a regulatory mechanism by which m^6^A influences circRNA-mediated immunomodulation. Therefore, m^6^A modification on circRNA inhibits innate immunity (Figure 4I) [95]. Numerous studies suggest that the crosstalk between circRNA and m^6^A modification should be considered simultaneously for their roles in disease regulation.

### 5.2. The Regulatory Role of Crosstalk Between m^6^A and Other Epigenetic Factors in Central Nervous System Development and Diseases

In recent years, extensive interactions between m^6^A RNA modification and other epigenetic mechanisms have been identified, which collaboratively regulate gene expression during central nervous system development and contribute to the pathogenesis of various neurological disorders (Table 1).

#### 5.2.1. Neurogenesis Disorder

During embryonic neural development, the distortion of cell-type diversity and the abnormal assembly of circuits and higher-level structures can lead to the occurrence of many mental disorders [96,97]. NSCs are a type of cell with division potential and self-renewal ability, which can produce various types of nerve tissue cells through asymmetric division. When METTL14 is deficient in embryonic NSCs, the downregulation of m^6^A increases the stability of histone acetyltransferase *Crebbp* and *Ep300* mRNA, promotes H3K27ac, inhibits proliferation-related genes, and activates the expression of differentiation-related genes, leading to the loss of NSCs’ ground state and inducing embryonic neurodevelopmental disorders [98]. As the basic structural and functional unit of the nervous system, the normal development of neurons is closely related to the construction of the central nervous system. It has been shown that mRNA in the neuronal cell body can be transported to dendrites and axons, and new proteins can be synthesized through mRNA local dynamic translation to regulate neuronal development and the correct establishment of neural networks. Related studies have found that lncRNA Dubr interacts with m^6^A-binding proteins YTHDF1 and YTHDF3 through m^6^A modification sites. The knockdown of Dubr or mutation of m^6^A sites in Dubr can accelerate the degradation of YTHDF1 and YTHDF3 proteins, affecting the mRNA translation of *Calmodulin* and *Tau* genes related to neuronal development, thereby inhibiting the axon growth of sensory neurons and the correct migration of cortical neurons [12]. In addition, m^6^A-mediated histone regulation also plays an important role in adult neurogenesis and neuronal development. The silencing of METTL3 in adult neural stem cells (ANSC) leads to proliferation inhibition and promotes ANSC differentiation towards the glial cell lineage, affecting the morphological maturation of newborn neurons. The downregulation of m^6^A levels induced by METTL3 deficiency is associated with the inhibition of histone methyltransferase EZH2 protein levels and H3K27me3, ultimately affecting the expression of genes related to proliferation, cell cycle progression, and neuronal development in ANSCs. The overexpression of EZH2 can repair the proliferation and neuronal development defects induced by METTL3 knockout in ANSCs [13]. Therefore, the crosstalk between m^6^A and epigenetic regulatory factors such as histone modification and lncRNA may be one of the mechanisms leading to neurodevelopmental disorders.

#### 5.2.2. Depression

Depression is one of the most common mental disorders worldwide and is characterized by emotional dysfunction [99,100,101]. Impaired synaptic plasticity is an important neuropathological basis for depression, which is usually associated with the dysregulation of relevant functional genes [102,103,104]. Studies on a chronic unpredictable mild stress (CUMS) rat model found that depression-like behavior induced by impaired synaptic plasticity may be due to the hypomethylation of the FTO promoter caused by the low expression of DNA methyltransferases Dnmt1 and Dnmt3a, affecting the expression of plasticity-related genes, and, thus, regulating depressive behavior [58]. It is known that patients with depression exhibit symptoms such as cognitive, emotional, memory, and neurogenic impairments [105], for which improving cognitive ability is a major goal of depression management [106]. Based on this, Niu et al. revealed that METTL3 is highly expressed in CUMS rats, and its silencing alleviates cognitive deficits by promoting the m^6^A-mediated processing and maturation of miR-221-3p through DGCR8, which, in turn, suppresses the expression of GRB2-associated binding protein 1 (Gab1)—a key regulator of synaptic plasticity, inflammation, oxidative stress, and apoptosis in the brain—thereby exacerbating cognitive impairment in CUMS models [107]. Furthermore, a survey of the Chinese Han population showed an association between ALKBH5 and severe depression, increasing the possibility of a high-risk correlation between ALKBH5 and this emotional disorder. Animal studies found an increased expression of ALKBH5 and depression-like behavior in mice subjected to chronic unpredictable stress. Mechanistic studies showed that CUMS caused the abnormal expression of circRNA STAG1, leading to the increased transport of ALKBH5 into the nucleus and the downregulation of the m^6^A modification of fatty acid amide hydrolase (FAAH) in astrocytes, promoting FAAH stability and inducing depression-like behavior [108]. These results suggest that there is extensive crosstalk between m^6^A, DNA methylation, miRNA, circRNA, and other epigenetic mechanisms that regulate the pathogenesis of depression, which may become a new target for depression treatment.

#### 5.2.3. Glioblastoma

Glioblastoma (GBM) is the most common and malignant primary intracranial tumor, characterized by high heterogeneity and poor prognosis [109,110]. However, epigenetic analysis has shown that dysregulated epigenetic mechanisms are involved in the formation of GBM [111], including crosstalk between various epigenetic factors. For example, crosstalk between m^6^A and histone modification can affect GBM resistance to therapy. The treatment of GBM cells with temozolomide (TMZ) has been reported to induce increased m^6^A modification in specific RNA transcripts, which indirectly affects the expression of histone modification enzymes (e.g., those regulating H3K27ac), leading to changes in chromatin accessibility, transcriptional plasticity, and therapy resistance [112]. Conversely, the crosstalk between m^6^A and histone modification is also a molecular mechanism for GBM treatment. H3K9 demethylase JMJD1C promotes the demethylation of the miR-302a promoter region to increase the expression of miR-302a, which negatively regulates METTL3 and inhibits GBM growth [113]. Furthermore, crosstalk between m^6^A and lncRNA promotes the proliferation of GBM. The m^6^A binding protein IGF2BP2 can recognize modification sites on lncRNA CASC9 (cancer susceptibility 9) and enhance its stability, thereby increasing the stability of hexokinase 2 mRNA, which is beneficial for aerobic glycolysis in GBM and promotes GBM proliferation [114]. In addition, studies have found that m^6^A-modified lncRNAs can predict the prognosis of GBM, such as lncRNA, MIR9-3HG, and LINC00515 as protective genes and LINC00900 and MIR155HG as risk genes [115]. Therefore, based on the interaction between m^6^A and lncRNA, m^6^A-modified lncRNA GBM risk prediction models have been established, providing new ideas for the treatment of GBM. These include the risk model of m^6^A-modified lncRNA RP11-552D4.1, which is associated with the immune status, immune suppression biomarkers, and chemosensitivity of GBM patients [116]. The GBM prognosis model constructed by m^6^A-modified lncRNAs AC005229.3, SOX21-AS1, AL133523.1, and AC004847.1 is used to predict and evaluate the survival rate of GBM patients [117].

#### 5.2.4. Other Diseases

In other CNS diseases, there is also crosstalk between m^6^A and other epigenetic regulatory factors. In autism, METTL3 stabilizes the expression of lncRNA MALAT1 by upregulating its m^6^A modification, which facilitates MALAT1 to recruit DNA methyltransferases DNMT1, DNMT3A, and DNMT3B to the promoter region of SFRP2, promoting SFRP2 methylation and reducing its expression, ultimately leading to Wnt/β-catenin signaling pathway inhibition, relieving hippocampal neuron apoptosis and autism-like symptoms [118]. In neurodegenerative diseases, the investigation of why paraquat (PQ) increases the risk of Parkinson’s disease (PD) found that PQ induces the m^6^A modification of lncRNA CDC5L and lncRNA STAT3, affecting their expression, increasing intracellular ROS production, affecting autophagy-related biological functions, and, ultimately, causing damage to and the death of dopaminergic neurons, leading to the onset of PD [119,120]. In stroke research, the currently successful in vitro stroke model is the oxygen and glucose deprivation/reoxygenation (OGD/R) protocol for neurons, which causes oxidative damage to surrounding neurons through ischemia–reperfusion [121,122]. Studies have found that OGD/R may induce neuronal cell apoptosis by increasing the m^6^A of lncRNA D63785 mediated by METTL3, downregulating the expression of LncD63585 and regulating the accumulation of miR-422a [123]. In conclusion, the occurrence of central nervous system diseases is not only regulated by a single epigenetic modification, but may involve the reprogramming of multiple epigenetic factors, so it is necessary to broaden the research field, explore new mechanisms, and discover new targets.

**Table 1:** Crosstalk mechanisms between m^6^A and other epigenetic regulatory factors in regulating central nervous system development and disease occurrence.

Categories	m^6^A Related Enzymes	Categories of Epigenetics	Related Components	Mechanisms	Biological Influence	References
Neurodevelopmental disorders	METTL14	Histone modification	*Crebbp* and *Ep300*	Upregulated H3K27ac level, inhibited proliferation genes, and activated differentiation genes	Disordered NSC ground state	[98]
	METTL3		EZH2	Inhibited H3K27me3 level; regulated P53 signaling pathway	Promoted ANSC to glial cell line; affected the development of newborn neurons	[13]
	YTHDF1YTHDF3	ncRNA	LncRNA Dubr	Accelerated degradation of YTHDF1 and YTHDF3; affected translation of Calmodulin and Tau	Inhibited axon growth; affected cortical neuron migration	[12]
Depression	FTO	DNA methylation	Dnmt1and Dnmt3a	Downregulated FTO expression	Influenced plasticity-related gene expression	[58]
	METTL3	ncRNA	pri-miR-221	Upregulated miR-221-3p; inhibited Gab1 expression	Induced cognitive impairment	[107]
	ALKBH5		circRNA STAG1	Reduced ALKBH5 level; promoted FAAH degradation	Induced depression-like behavior	[108]
Glioblastoma	METTL3	Histone modification	EZH2	Increased H3K27ac level; degraded nonsense-mediated mRNA	Increased drug resistance	[112]
			JMJD1C	Inhibited SOCS2 expression	Inhibited GBM growth	[113]
	IGF2BP2	ncRNA	lncRNA CASC9	Increased the stability of HK2; promoted aerobic sugar degradation	Promoted GBM proliferation	[114]
	—		lncRNA RP11-552D4.1	—	Reflected immune infiltration disorders; predicted GBM risk	[116]
	—		lncRNA AC005229.3/SOX21- AS1/AL133523.1/ AC004847.1	—	Reflected immune response function; predicted GBM prognosis	[117]
Autism	METTL3	ncRNA	lncRNA MALAT1	Downregulated SFRP2 expression; inhibited Wnt/β-catenin signaling pathway	Reduced autism-like symptoms and hippocampal neuronal apoptosis	[118]
Parkinson’s disease	—	ncRNA	lncRNA CDC5L and lncRNA STAT3	Increased ROS production; enhanced autophagy	Induced dopaminergic neuron damage and death	[119, 120]
Stroke	METTL3	ncRNA	LncRNA D63785	Increased the accumulation of miR-422a	Induced neuronal apoptosis	[123]

## 6. Therapeutic Potential of Targeting Crosstalk Between m^6^A and Other Epigenetic Modulators

In recent years, drug development targeting m^6^A regulators has made initial progress, providing both theoretical and technical foundations for intervening in their crosstalk with other epigenetic mechanisms. Emerging evidence suggests that m^6^A modifications form a coordinated regulatory network with DNA methylation, histone modifications, and non-coding RNAs during various physiological and pathological processes. Therefore, the combinatorial targeting of these pathways may enhance therapeutic efficacy and overcome the limitations of single-target interventions.

Several small-molecule compounds have been developed to target m^6^A regulators. Rhein was the first identified natural inhibitor of FTO, followed by the development of more selective and potent inhibitors such as FB23, FB23-2, CS1, CS2, and Bisantrene, which have shown the ability to inhibit leukemia cell proliferation or reduce neuronal apoptosis. The METTL3 inhibitor STM2457 has demonstrated anti-tumor activity in multiple cancer types and is currently in early-phase clinical trials [124]. The inhibitors of ALKBH5 are still in the early stages of investigation, but preliminary evidence indicates their potential role in diseases such as glioblastoma [125,126].

Although most of these compounds are currently explored within the oncology field, the growing literature suggests that targeting m^6^A regulators may also indirectly influence DNA methylation (e.g., DNMT1 expression), histone deacetylation (e.g., HDACs), or the stability and expression of miRNAs/lncRNAs, thereby facilitating the integrated modulation of multiple epigenetic mechanisms. For instance, the FTO inhibitor FB23-2 can modulate the expression of neuroinflammation-associated miRNAs (e.g., miR-146a) through m^6^A regulation, thereby affecting downstream TLR signaling and histone acetylation [127]. METTL3-mediated m^6^A modification has been shown to promote the stability of EZH2, a histone methyltransferase, influencing H3K27me3 levels and suggesting direct crosstalk between m^6^A and histone modifications with potential therapeutic relevance [128].

Furthermore, the development of multi-target epigenetic inhibitors is emerging as a promising strategy. Some novel compounds can simultaneously interfere with m^6^A regulatory proteins and other epigenetic factors (such as DNMTs and HDACs), enabling combinatorial intervention approaches. These agents have demonstrated neuroprotective effects in models of neurodegenerative diseases [129].

In summary, although no clinical-grade drugs currently exist that specifically target the crosstalk between m^6^A and other epigenetic regulators, several candidate molecules have demonstrated synergistic effects in preclinical studies. However, most of these compounds remain at the in vitro or early in vivo research stages, with limited clinical validation. Existing clinical trials—such as those involving the METTL3 inhibitor STM2457—are primarily focused on oncology, and systematic in vivo evaluation in neurological disease models is still lacking. These limitations highlight the need for further pharmacokinetic optimization, toxicity profiling, and long-term safety assessment to support future translational applications. Collectively, these findings lay the groundwork for the development of multi-faceted network-based epigenetic therapies for neurological disorders.

## 7. Summary and Outlook

Advancements in sequencing technologies have progressively uncovered the physiological roles of m^6^A modification, including its regulation of RNA splicing, nuclear export, translation, and stability. Concurrently, growing evidence has illuminated the interplay between m^6^A and other epigenetic regulators—such as DNA methylation, histone modifications, and long non-coding RNAs—shedding light on the mechanisms of epigenetic reprogramming. Given the remarkable complexity, heterogeneity, and plasticity of the nervous system, elucidating the crosstalk between m^6^A and other epigenetic pathways may offer novel insights for enhancing therapeutic strategies in central nervous system disorders.

From a translational perspective, therapeutic strategies that target m^6^A regulatory proteins hold promise for the development of novel pharmacological interventions against a range of CNS disorders, including Alzheimer’s disease, major depressive disorder, and post-stroke cognitive impairment. On the one hand, the pivotal role of m^6^A in neurogenesis, synaptic plasticity, and neuroinflammatory modulation makes it an attractive target for the precision treatment of neuropsychiatric diseases. On the other hand, the extensive crosstalk between m^6^A, DNA methylation, and histone modifications provides a theoretical rationale for combined epigenetic targeting. Future investigations should integrate cutting-edge technologies such as single-cell sequencing, spatial transcriptomics, and AI-driven drug discovery platforms to identify cell-type-specific m^6^A nodes and construct highly precise epigenetic intervention models. At the drug development level, high-throughput screening coupled with structure-guided optimization will be essential to improve the brain penetrance and target selectivity of enzyme inhibitors—for example, those directed against FTO or METTL3—thereby enhancing their therapeutic potential in CNS diseases.

In summary, an in-depth understanding of m^6^A regulation and its epigenetic crosstalk not only promises breakthroughs in the mechanistic elucidation of neurological disorders, but also furnishes a theoretical and practical foundation for developing clinically translatable precision epigenetic interventions.

## Data Availability

Not applicable.
